# IMPERATIVE: Harnessing male peer networks to enhance engagement with HIV prevention: A large-scale cluster randomised trial to increase HIV self-testing and PrEP uptake among men in Eastern Zimbabwe

**DOI:** 10.21203/rs.3.rs-9814858/v1

**Published:** 2026-06-05

**Authors:** Paul Mee, Wilfred Otambo, Morten Skovdal, Guy Harling, Michael Pickles, Adrian Dobra, Timothy Hallett, Till Bärnighausen, Maureen McGowan, Maxime Inghels, Phyllis Mandizvidza, Louisa Moorhouse, Blessing Tsenesa, Rufurwokuda Maswera, Freedom Dzamatira, Simon Gregson, Constance Nyamukapa, Frank Tanser

**Affiliations:** University of Lincoln; Stellenbosch University; University of Copenhagen; University College London; Imperial College London; University of Washington; Imperial College London; Heidelberg University; Heidelberg University; University of Lincoln; Biomedical Research and Training Institute; Imperial College London; Biomedical Research and Training Institute; Biomedical Research and Training Institute; Biomedical Research and Training Institute; Imperial College London; Imperial College London; Stellenbosch University

**Keywords:** HIV self-testing, pre-exposure prophylaxis, PrEP, Peer networks, Men, Zimbabwe, Randomised controlled trial, Implementation science

## Abstract

**Background:**

Engaging men in HIV prevention remains a critical challenge in sub-Saharan Africa. While HIV self-testing (HIVST) and pre-exposure prophylaxis (PrEP) are efficacious prevention methods, uptake among men remains low. Peer network distribution of HIVST has shown promise, but no large-scale studies have evaluated its impact on PrEP uptake combined with mobile phone support, especially among men. This protocol describes a cluster randomised controlled trial evaluating a multi-component intervention to increase HIVST use and PrEP uptake among men through peer network distribution with enhanced risk perception counselling and telephone support.

**Methods/Design:**

This trial will be implemented in 44 clusters across eight sites in Manicaland Province, Eastern Zimbabwe. Eligible men aged 18 and above will be recruited through peer network distribution initiated by primary distributors. The intervention includes: (1) HIVST kit distribution through male peer networks, (2) toll-free helpline for pre- and post-test support, (3) SMS-based self-administered HIV risk assessment, and (4) facilitated linkage to confirmatory testing and PrEP at local clinics, including incentivisation and compensation. The primary outcome is the proportion of men initiating PrEP. Secondary outcomes include ART initiation, clinic-based HIV testing rates, and PrEP retention at one-month follow-up. The study is powered to detect an increase from 2% to 8.5% in PrEP initiation. A comprehensive process evaluation will assess implementation fidelity and network characteristics affecting outcomes. Mathematical modelling will project population-level impact and cost-effectiveness.

**Discussion:**

This will be the first large-scale randomised trial to evaluate whether peer network-based HIVST distribution combined with mobile health support and improved risk perception increases PrEP uptake among men in a high HIV-burden setting. If successful, this scalable intervention could inform national HIV prevention programmes across sub-Saharan Africa seeking to improve male engagement with prevention services.

**Trial registration:**

ClinicalTrials.gov NCT06370923. Registered on 12-Apr-2024.

## Structured summary {1b}

**Table:** 

Item	Description
Primary Registry and Trial Identifying Number {4}	ClinicalTrials.gov NCT06370923. Registered on 12-Apr-2024. https://clinicaltrials.gov/study/NCT06370923
Secondary Identifying Numbers	
Source(s) of Monetary or Material Support	This study is funded by the United States National Institute of Health through the National Institute of Mental Health [R01MH133488]. The funder had no role in study design, data collection, analysis, interpretation, or manuscript preparation.
Primary Sponsor and contact information {3b}	Stellenbosch University, Stellenbosch, South Africa
Role of sponsor and funder {3c}	Sponsor and funder have no input into the trial design and operations.
Contact for Public Queries	Frank Tanser ftanser@sun.ac.za South African Centre for Epidemiological Modelling and Analysis (SACEMA), Centre for Epidemic Response and Innovation (CERI), School for Data Science and Computational Thinking, Stellenbosch University, Cape Town, South Africa
Contact for Scientific Queries	Frank Tanser ftanser@sun.ac.za South African Centre for Epidemiological Modelling and Analysis (SACEMA), Centre for Epidemic Response and Innovation (CERI), School for Data Science and Computational Thinking, Stellenbosch University, Cape Town, South Africa
Public Title	IMPERATIVE
Scientific title	Harnessing male peer networks to enhance engagement with HIV prevention: A large-scale cluster randomised trial to increase HIV self-testing and PrEP uptake among men in Eastern Zimbabwe
Countries of Recruitment	Zimbabwe
Health Condition(s) or Problem(s) Studied	HIV prevention, HIV testing, HIV self-testing
Intervention(s)	The intervention comprises multiple integrated components delivered sequentially through male peer networks: **Primary distributor recruitment and training** Primary distributors will be identified by the research team through engagement with local key informants. Desired characteristics include strong local peer networks and community standing. Following informed consent, distributors will receive a pack containing four HIVST kits (one for personal use, three for peer distribution) along with training on HIVST use and interpretation, post-test confirmatory testing procedures, and HIV prevention methods including PrEP availability. Basic demographic data (name, age, mobile phone number) will be collected for distributors and intended recipients, along with unique kit pack identification numbers. **Peer-to-peer HIVST distribution** Distributors will distribute HIVST kits to peers within their cluster within seven days. After seven days, the helpline will contact both distributors and recipients to: (1) confirm kit receipt and use, (2) collect behavioural HIV risk data, and (3) encourage clinic attendance for confirmatory testing. Recipients who consent will be enrolled in the study and invited to become secondary distributors. Distributors will receive 1 US Dollar (USD) for each kit successfully distributed and 1USD for attending confirmatory testing. **Network expansion through community facilities** Men wishing to become distributors can obtain HIVST packs (three kits per pack) from designated community facilities (e.g. shops, pharmacies, hairdressers, beer halls). Facility owners will verify eligibility using a real-time database. Successful distributors may collect up to two packs (maximum six kits total). This community facility-based distribution model has proven successful in other sub-Saharan African settings. **Toll-free helpline support** A toll-free helpline staffed by trained counsellors will provide: (1) pre-test counselling, (2) post-test support and interpretation assistance, (3) facilitation of clinic appointments for confirmatory testing, and (4) adherence support for those initiating PrEP or ART. The helpline will conduct regular follow-up calls at defined intervals to support participants through the prevention cascade. **SMS-based HIV risk assessment** Recipients will receive an SMS-based HIV risk assessment tool allowing them to self-assess infection risk based on behavioural factors. This assessment will provide personalised risk feedback and PrEP eligibility screening to facilitate rapid linkage to prevention services.
Key Inclusion and Exclusion Criteria	**Inclusion criteria:** Male Aged 18 years and above Resident within defined cluster boundaries Expected to remain resident for the study duration **Exclusion criteria:** Currently taking PrEP at baseline Known HIV-positive status (men testing positive during the trial will be referred to ART services) In addition, people living with HIV and those taking PrEP at baseline can enrol as distributors and recruit peers but are excluded from outcomes.
Study Type	This is a prospectively registered, two-arm matched-cluster randomised controlled trial with assessor masking and intention-to-treat analysis. The study is being implemented in eight sites in Manicaland Province, Eastern Zimbabwe. Within each site, clusters based on village boundaries will be randomly allocated to intervention or control arms in a public randomisation event to maximise community acceptance.
Date of First Enrollment (planned)	16th October 2024
Sample Size	3,590
Primary outcome(s)	Proportion of men aged 18 and above who initiate PrEP during the trial period, comparing intervention and control clusters.
Key Secondary outcome(s)	Proportion of men aged 18 and above initiating antiretroviral therapy (ART) Proportion of men aged 18 and above initiating either ART or PrEP (combined outcome) Proportion of men aged 18 and above receiving clinic-based HIV testing Proportion of men tested at clinic who are diagnosed HIV-positive Proportion of men tested at clinic who used HIVST in the previous three months Proportion of men initiating PrEP who return for first follow-up visit Proportion of men initiating ART who return for first follow-up visit.
Ethics Review	Medical Research Council of Zimbabwe Reference No: MRCZ/A/3098 and the Stellenbosch University Health Research Ethics Committee, Reference No: M23/03/009
Individual Trial Participant Data sharing statement	De-identified data will be made available to the research community upon reasonable request after final outcomes and subsequent analyses have been published, subject to data sharing agreements protecting participant confidentiality.

## Protocol version {2}

September 2024 (version #3.1 16th Sept 2024). Any protocol amendments will be communicated to investigators, Human Ethics Research Committees, Trial Steering Committee, Data Safety and Monitoring Board, trial participants, and trial registries.

## Introduction

### Background and rationale {9a}

Effectively engaging men in HIV prevention remains one of the greatest challenges to achieving epidemic control in sub-Saharan Africa (SSA) [1–3]. Men in SSA face multiple gender-specific barriers to accessing HIV testing and care services, including rigid masculinity norms, poor risk perception, stigma, and limited clinic accessibility [4, 5]. As a result, men are less likely than women to know their HIV status, initiate treatment, and remain engaged with care [1, 2, 6]. This suboptimal engagement not only increases men's risk of HIV acquisition but also drives new infections in women [2].

HIV self-testing (HIVST) addresses several facility-based access barriers and has been shown to be acceptable and feasible for distribution through male peer networks in SSA [7–9]. The distribution of HIVST through peer networks can increase penetration of health interventions to hard-to-reach individuals [10].

Oral pre-exposure prophylaxis (PrEP) can substantially reduce HIV incidence in high-risk populations, with demonstrated efficacy of up to 75% reduction in clinical trials [11, 12]. However, uptake of oral PrEP among men in SSA remains low, despite evidence of high demand [13]. Our hypothesis for this study is that men using HIV self-test kits (HIVST) would be more likely to engage with PrEP. Previous studies have explored the use of social network and community-based HIVST distribution models to increase PrEP uptake [14] [15]. It was found in the Manicaland study site that whilst approximately 60% of men were aware of HIVST, fewer than 6% had used the kits.[16]

Several efficacious HIV prevention strategies exist in isolation: HIVST has been integrated into national HIV strategies across SSA [7]; peer encouragement increases men's willingness to test [8, 17]; and mobile phone-based interventions can improve HIV care engagement and adherence [18, 19]. However, a significant knowledge gap exists on how to effectively combine these approaches at scale to increase PrEP uptake among men.

HIV prevention cascades (HPCs) provide a framework to identify multilevel barriers that prevent people from fully benefiting from prevention methods [20–22] and inform targets for interventions to reduce such barriers. Applied to men in Manicaland, Zimbabwe, the HPC revealed multiple barriers to PrEP uptake, including low knowledge, poor risk perception, and extremely limited access [16].

### Explanation for the choice of comparator {9b}

Participants in the control and intervention arms will receive the current standard of care for identifying those at risk of HIV infection which may include targeted community outreach activities by community health workers (CHW) or Non-Governmental Organisations. This will enable the trial to assess the additional impact of the peer distribution of HIVST which will only occur within clusters in the intervention arm.

### Objectives {10}

The primary objective of this cluster randomised controlled trial is to determine the impact of HIVST distribution through male peer networks, combined with mobile phone-based support and improved risk perception, on PrEP uptake among men in Eastern Zimbabwe.

The specific aims of the study are:

1. To evaluate the effectiveness of a peer network-based HIVST distribution intervention with mobile support on PrEP initiation among men aged 18 and above
2. To conduct a comprehensive process evaluation characterizing peer networks, implementation fidelity, and mechanisms underlying trial effectiveness
3. To quantify population-level impact and cost-effectiveness of the intervention using mathematical modelling

## Methods: Patient and public involvement, and trial design

### Patient and public involvement {11}

A ‘Community Advisory Board’ (CAB) will be established consisting of community members from each of the eight study sites. The CAB will serve as an advisory group providing the perspectives of local community members to the study and supporting all stages of the study. CAB members will be selected to represent a range of different perspectives including different male/female and different age-groups. The CAB will meet every 3 months throughout the study. Any CAB members who are legal minors will be accompanied during travel to and from CAB meetings and at the meetings themselves by community elders from their home areas. Additionally, other community members will be engaged with through local engagement throughout the study.

### Trial design {12}

This is a prospectively registered, two-arm matched-cluster randomised controlled trial with assessor masking and intention-to-treat analysis. The study is being implemented in eight sites in Manicaland Province, Eastern Zimbabwe. Within each site, clusters based on village boundaries will be randomly allocated to intervention or control arms in a public randomisation event to maximise community acceptance.

The study design leverages infrastructure from a 30-year program of HIV surveillance and behavioural research in this well-characterized population cohort [23]. The intervention implementation considers feedback from participatory ‘theory-of-change’ workshops with key community stakeholders [24]. This article adheres to the Standard Protocol Items: Recommendations for Interventional Trials (SPIRIT) 2025 guidance [25]

## Methods: Participants, interventions and outcomes

### Trial setting {13}

**Figure 1:**
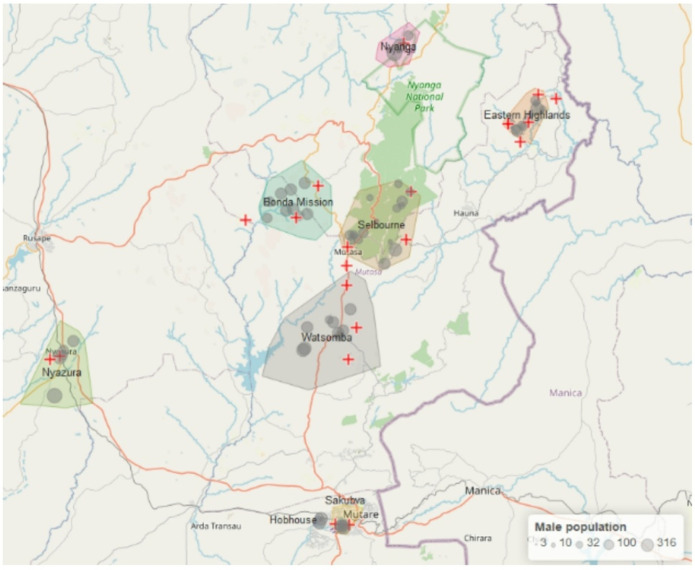
Map of the study sites included in the study. These sites represent the 5 major socioeconomic strata in Manicaland; small towns (Nyanga and Nyazura), agricultural estates (Eastern Highlands and Selbourne), roadside settlements (Watsomba), subsistence farming areas (Bonda) and urban areas (Hobhouse and Sakubva), Red crosses indicate the location of the study clinics.

The study is being conducted in Manicaland Province, Eastern Zimbabwe, covering an area of 36,459 km^2^ with a population of approximately 1.75 million. Study sites represent five major socioeconomic strata: small towns, agricultural estates, roadside settlements, subsistence farming areas, and large urban areas (Fig. 1) [23].

Despite recent declines in new infections[26], data from 2019 showed that HIV prevalence remained high, peaking at 27% for women aged 45–49 years and 28% for men aged 50–54 years [27]. Local healthcare is provided through a network of public sector primary care clinics, with some operating as public-private partnerships. Oral PrEP has been recently adopted as a key component of Zimbabwe's national AIDS response and is available free of charge at all local clinics. The expected characteristics and inclusion and exclusion criteria for trial participants is summarized in Table 1.

## Characteristics of the people who are needed for the trial

### Eligibility criteria for participants {14a}

#### Inclusion criteria:

- Male
- Aged 18 years and above
- Resident within defined cluster boundaries
- Expected to remain resident for the study duration

#### Exclusion criteria:

- Currently taking PrEP at baseline
- Known HIV-positive status (men testing positive during the trial will be referred to ART services)

In addition, people living with HIV and those taking PrEP at baseline can enrol as distributors and recruit peers but are excluded from outcomes.

### Eligibility criteria for sites and those delivering interventions {14b}

The sites for the study were drawn from Manicaland Centre for Public Health surveillance site. Interventions will be delivered by the Centre staff and trained study nurses.

### Who will take informed consent? {32a}

Trained research assistants holding Good Clinical Practice (GCP) certificates will obtain informed consent from primary distributors on a one-to-one basis in a private setting. Consent will be conducted in either Shona or English according to participant preference. Research assistants will describe the study including potential risks and benefits, the right to withdraw without loss of healthcare entitlements, and will address any questions.

For HIVST kit recipients, informed consent will be completed remotely by phone. Consent decisions will be recorded on a separate mobile device, with participants confirming their name, their understanding of the study, and their willingness to participate. Participants will also receive a printed copy of the informed consent form within the HIVST kit insert. Consent for clinical data collection will be obtained by GCP trained study nurses based at the clinics.

### Additional consent provisions for collection and use of participant data and biological specimens {32b}

All data and biological specimens collected will be used solely for the purposes outlined in this protocol and are covered under the primary informed consent process. Should additional sub studies be conducted, separate informed consent will be obtained from all relevant participants prior to participation.

## Intervention and comparator

### Intervention and comparator description {15a}

#### Intervention arm: Peer network HIVST distribution with mobile support

**Figure 2:**
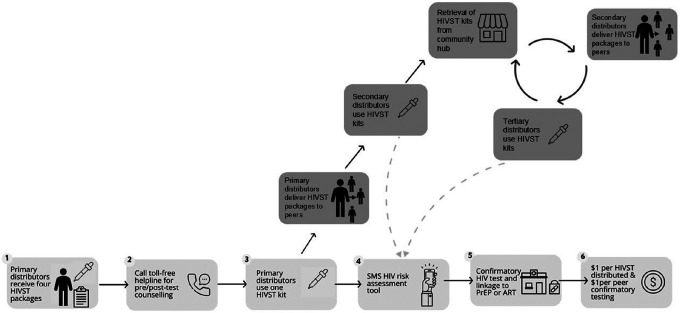
Overview of the components of the intervention arm of the IMPERATIVE study

The intervention comprises multiple integrated components delivered sequentially through male peer networks. The components of the intervention arm of the trial are summarised in Fig. 2.

#### Primary distributor recruitment and training

Primary distributors will be identified by the research team through engagement with local key informants. Desired characteristics include strong local peer networks and community standing. Following informed consent, distributors will receive a pack containing four HIVST kits (one for personal use, three for peer distribution) along with training on HIVST use and interpretation, post-test confirmatory testing procedures, and HIV prevention methods including PrEP availability. Basic demographic data (name, age, mobile phone number) will be collected for distributors and intended recipients, along with unique kit pack identification numbers.

#### Peer-to-peer HIVST distribution

Distributors will distribute HIVST kits to peers within their cluster within seven days. After seven days, the helpline will contact both distributors and recipients to: (1) confirm kit receipt and use, (2) collect behavioural HIV risk data, and (3) encourage clinic attendance for confirmatory testing. Distributors will also be asked for the names and contact details for those they gave the HIVST kits to. Recipients who consent will be enrolled in the study and invited to become secondary distributors. Distributors will receive 1 US Dollar (USD) for each kit successfully distributed.

#### HIVST kit supply through community facilities

Community hubs will serve as accessible, community-based distribution points from which men who have agreed to become distributors can collect HIVST kit packs. Suitable hub locations, which may include shops, bottle stores, and beer halls, will be identified through consultation with the Community Advisory Board and community members to ensure they represent places of convenience that men are comfortable approaching. Hub operators will enter into a formal agreement with the study team, receive training in study procedures, and be supplied with a lockable insulated storage box, disbursement forms, and a toll-free helpline number for support and restocking requests. Men who have registered their wish to become distributors via the helpline will attend their nearest hub, where the operator will verify their identity and eligibility against a real-time SMS-accessible database before issuing a pack of three HIVST kits; hub operators will receive compensation of 1 USD per pack successfully distributed. This model of HIVST distribution through community-based hubs has been demonstrated to be effective in other sub-Saharan African settings [28].

#### Toll-free helpline support

A toll-free helpline staffed by trained counsellors will provide: (1) pre-test counselling, (2) post-test support and interpretation assistance, (3) facilitation of clinic appointments for confirmatory testing, and (4) adherence support for those initiating PrEP or ART. The helpline will conduct regular follow-up calls at defined intervals to support participants through the prevention cascade.

#### SMS-based HIV risk assessment

Recipients will receive an SMS-based HIV risk assessment tool to evaluate their personal infection risk based on behavioural factors. The tool uses questions drawn directly from the national HIV PrEP eligibility screening criteria, providing standardised, personalised risk feedback and a clear indication of PrEP eligibility. This is intended to facilitate rapid linkage to prevention services for those who are eligible. Clinic staff will be briefed on the tool, enabling recipients to present their results at the clinic to support their PrEP eligibility assessment.

#### Study Clinics

All clinics and hospitals providing HIV services within the catchment areas of the study clusters were identified and included. Staff responsible for HIV testing, prevention, and treatment services at each clinic will be trained in all relevant study protocols and in Good Clinical Practice (GCP). Following completion of HIVST distribution in each cluster, clinic staff will collect data from all men attending for HIV testing over a follow-up period of up to three months. Men attending from an intervention cluster will be included in the cluster-level outcomes regardless of whether they personally participate in the intervention.

#### Clinic linkage and PrEP initiation

Participants will receive a referral form with a unique barcode for return to clinics during confirmatory testing. Based on self-reported HIVST results (reactive/non-reactive), individuals will be screened for PrEP or ART eligibility. Screening outcomes will be recorded a clinic information form by the clinic staff. All individuals attending clinic for confirmatory testing will receive 1USD reimbursement.

#### Follow-up and adherence monitoring

Individuals initiating PrEP or ART in the intervention arm will be contacted monthly for six months to provide adherence support and monitor continuation. In the control arm follow-up and adherence will be assessed using clinic records. At six months, those self-reporting continued PrEP/ART use will provide dried blood spots for analysis of intracellular c (TFV-DP) levels to objectively verify adherence [29].

#### Incentive structure

To encourage distribution, primary distributors will receive 1USD electronically for each kit successfully distributed to a peer who is contacted by the helpline (regardless of whether the peer uses the kit or enrols as a distributor). An additional 1USD remuneration will be paid to participants who attend the clinic and take a confirmatory HIV test. Community facility owners will receive 1USD for each pack distributed. These modest incentives are designed to offset time and mobile phone costs without creating coercive inducements.

#### Control arm

Standard of care Community outreach, HIV testing and PrEP services will remain available through existing government health facilities and distribution of HIVST by community health workers in all clusters. This will be the control in this study. Men in control clusters will be followed for outcome measurement at the same time points as intervention clusters. However, the collection of dry blood spots to assess PrEP adherence will only be carried out for men in the intervention arm. The geographic location of the residence of men from control clusters will be determined by the clinic staff by referral to detailed maps.

### Criteria for discontinuing or modifying allocated intervention/comparator {15b}

Serious adverse events will be reported to the institutional review boards within Stellenbosch University Medical Research Ethics Committee and the Medical Research Council of Zimbabwe. An adverse event log will be maintained and reviewed regularly by the Study Steering Committee.

### Strategies to improve adherence to intervention/comparator {15c}

Through the telephone helpline individuals will be encouraged to attend the clinic for a confirmatory HIV test and for regular follow-up appointments and to remain adherent to PrEP or ART if they have initiated prevention or treatment.

### Concomitant care permitted or prohibited during the trial {15d}

There are no concomitant care restrictions in this study.

### Ancillary and post-trial care {34}

All post-trial care will be provided through the public health services available to participants.

### Outcomes {16}

#### Primary outcome

The primary outcome is the proportion of men aged 18 and above who initiate PrEP during the trial period, comparing intervention and control clusters.

#### Secondary outcomes

- Proportion of men aged 18 and above initiating antiretroviral therapy (ART)
- Proportion of men aged 18 and above initiating either ART or PrEP (combined outcome)
- Proportion of men aged 18 and above receiving clinic-based HIV testing
- Proportion of men tested at clinic who are diagnosed HIV-positive
- Proportion of men tested at clinic who used HIVST in the previous three months
- Proportion of men initiating PrEP who return for first follow-up visit
- Proportion of men initiating ART who return for first follow-up visit

The male population aged over 18 in each cluster as measured in a household census carried out concurrently with the trial will be used to calculate the denominators used in calculating these proportions.

### Harms {17}

All adverse events related to study participation will be documented and reported to ethics committees in accordance with standard procedures. Potential risks and mitigation strategies include:

Several potential risks to participants have been identified and corresponding mitigation measures have been incorporated into the study design.

Confidentiality will be protected using secure data management systems, separation of identifiable data, staff training on confidentiality procedures, and the provision of anonymous feedback mechanisms.

Psychological distress arising from HIV testing will be addressed through access to trained counsellors via the study helpline, standard post-test counselling protocols, and referral pathways to appropriate support services where required.

The potential for social harm is of particular relevance to this study, as distributors and recipients of HIVST kits may experience stigma from community members if they are perceived to belong to peer networks associated with high-risk sexual behaviour. This risk will be mitigated through two complementary approaches. First, community engagement activities will reinforce the message that all men aged 18 and above are eligible to participate regardless of their behavioural risk profile or HIV status. Second, all contact between the study team and participants, whether in person or by telephone, will be conducted privately and confidentially. When contacting participants by phone, study staff will verify the participant’s identity and confirm that they are in a location where they are able to speak freely. If this is not the case, a follow-up call will be scheduled for a time when the participant is in a private setting.

Adverse effects associated with HIVST or PrEP use will be managed in accordance with standard clinical protocols at participating health facilities, with all adverse events documented and reported through the appropriate channels.

The risk of coercion arising from study incentives will be minimised by keeping incentives modest, set at 1USD, and by framing these explicitly as reimbursement for participants' time and costs rather than as payment for participation.

If a serious adverse event is reported, unblinding of an individual participant’s allocation may be undertaken if directed by the ethics committee.

### Participant timeline {18}

The study timeline for participants is shown in Table 2. Enrolment into the intervention arm will be conducted by study helpline staff and will occur within one month of the distributor receiving the HIVST kit pack. Enrolment into the control arm will be carried out by GCP trained nurses based at the study clinics.

Given the large geographic spread of clusters across the study site, recruitment will be conducted in four phases, with geographically proximate clusters grouped together within each phase. Each phase will have a recruitment window of six months, with an overall recruitment period of 24 months across all phases.

### Sample size {19}

Sample size calculations are based on detecting a programmatically meaningful difference in the primary outcome (PrEP initiation) between intervention and control arms. Assuming a baseline PrEP initiation rate of 2% in control clusters and targeting detection of an increase to 8.5% in intervention clusters, with 80% power (alpha = 0.05), we estimated a requirement of 22 clusters per arm.

Sample size calculations account for: (1) an intra-cluster correlation coefficient (ICC) estimated at 0.04, based on previous HIV incidence data from the study sites; (2) a mean cluster size of 81 men; (3) a coefficient of variation in cluster size of 0.15; and (4) an anticipated consent rate of 80% and retention rate of 80% at six months. The total estimated sample is 3,590 men (1,795 per arm) across 44 clusters.

### Recruitment {20}

Recruitment will proceed through multiple pathways:

- **Primary distributors**: Identified through engagement with local key informants (community leaders, health workers, established community groups) who can recommend men with strong local networks.
- **Peer network recruitment**: Men receiving HIVST kits from distributors will be contacted by the helpline and invited to enrol in the study.
- **Recruitment in control communities**: Men in control communities will be enrolled by trained nurses when they attend the clinic for HIV testing.

## Assignment of interventions: randomisation

### Sequence generation: who will generate the sequence {21a}

The randomisation sequence will be generated by the study statistician.

### Sequence generation: type of randomisation {21b}

All participants will be assigned to clusters consisting of one or more distinct villages, as described in the sample size section. Clusters will be randomly allocated to either the intervention or control arm. Allocation will be stratified by the five major socioeconomic strata present across Manicaland: small towns, agricultural estates, roadside settlements, subsistence farming areas, and urban areas, to ensure balance between strata in each arm. Within each study site, detailed maps will be used to create matched clusters of villages based on socioeconomic and geographic characteristics. Clusters will be pair-matched within sites to ensure balance prior to randomisation.

### Allocation concealment mechanism {22}

Allocation concealment is achieved through cluster-level randomisation occurring before individual participant recruitment, preventing selection bias based on treatment assignment. Cluster allocation will be performed only after all clusters have been fully defined and mapped.

### Implementation {23}

The study statistician will generate 100 acceptable randomisations (22 intervention and 22 control clusters), numbered 0 to 99. A public randomisation event will then be held to select which permutation to use and to determine which arm is designated as intervention or control.

## Assignment of interventions: blinding

### Who will be blinded {24a}

Due to the nature of the intervention, participants and intervention delivery staff cannot be blinded to cluster allocation. However, outcomes assessors and data analysts will be masked to cluster allocation during data collection and analysis wherever possible.

### How will be blinding be achieved {24b}

Those carrying out interim data analyses will not be provided with data indicating which trial arm trial participants were allocated to.

## Data collection and management

### Plans for assessment and collection of outcomes {25a}

#### Data collection methods

Data will be collected through multiple sources:

- **Distributor and recipient tracking**: Demographic data, contact information, and HIVST kit distribution information collected at enrolment by field staff using electronic data capture
- **Helpline records**: Call logs, behavioural HIV risk assessments, and support interactions documented in secure database
- **Clinic records**: HIV testing results, PrEP/ART initiation dates, follow-up visit attendance collected via referral forms with unique barcodes and routine clinic data
- **Biological specimens**: Dried blood spots collected at six months from participants in the intervention arm self-reporting PrEP/ART adherence for Tenofovir diphosphate (TFV-DP) analysis
- **Qualitative data**: Audio recordings of workshops, focus group discussions, interviews, and photovoice reflections

### Plans to promote participant retention and complete follow-up {25b}

Participant retention and complete follow-up are critical to the integrity of the trial, particularly given that men in Manicaland represent a mobile population. Multiple complementary strategies will be employed to minimise attrition. At enrolment, basic contact information including mobile phone numbers will be collected for all distributors and recipients, enabling proactive follow-up throughout the study period. The toll-free helpline will conduct scheduled follow-up calls at monthly intervals for all participants who have initiated PrEP or ART, providing both adherence support and an opportunity to maintain engagement with the study. Participants who have initiated PrEP or ART will be contacted monthly for six months, with a dry blood spot collection scheduled at six months to verify adherence objectively for those in the intervention arm. Financial reimbursement of 1USD will be provided to participants attending clinic for confirmatory testing, offsetting travel and time costs that represent common barriers to follow-up in this setting. Clinic staff will be trained in study procedures and will use referral forms with unique barcodes to track participant attendance and linkage, enabling the study team to identify and re-engage participants who have missed appointments. Where a participant cannot be reached, study staff will attempt contact through alternative means recorded at enrolment. These strategies are designed to achieve the anticipated retention rate of 80% at six months on which the sample size calculations are based.

### Data management {26}

All data will be collected electronically using secure tablets and mobile devices with password protection. Data will be uploaded daily to secure servers with automated backup systems. Data quality will be monitored through a combination of complementary procedures. Real-time validation checks will be applied during data entry, supported by weekly quality control reports and monthly data quality audits. These measures will be supplemented by regular cross-checking between data sources, including matching of helpline records against clinic referral forms.

Personal identifiers will be stored separately from study data using unique study IDs. Only authorised study staff will have access to identifiable data. All data will be stored on encrypted servers with access limited to designated team members. Paper documents will be stored in locked filing cabinets in secure offices.

Qualitative audio recordings will be transcribed, anonymised, and deleted following transcription.

### Confidentiality {33}

Participant confidentiality will be protected through a series of complementary measures. All documents and biological specimens will be identified using unique study IDs, with personal identifiers stored securely and separately from study data. Electronic systems will be password-protected with access restricted to authorised personnel, and paper records will be held in locked filing cabinets. All study staff will receive training on confidentiality protocols. Data sharing will be limited to aggregated, de-identified data, and access to identifiable information will be restricted to authorised study personnel where this is necessary for study conduct.

## Statistical methods

### Statistical methods for primary and secondary outcomes {27a}

#### Primary analysis

Analysis will be conducted on an intention-to-treat basis. For the primary outcome, cluster-level PrEP initiation proportions will be compared between intervention and control arms using regression models, adjusting for any baseline imbalances. We will perform statistical analyses at the individual level to model the probability of PrEP initiation using binary regression (logistic and probit) with fixed effects to adjust for age and sex, and random effects to adjust for clusters. In addition, we will perform statistical analyses at the cluster level with beta regression models for the cluster-level PrEP initiation proportions adjusted for cluster characteristics. Effect sizes will be reported with 95% confidence intervals. Results will be considered statistically significant if P < 0.05. Sensitivity analyses will explore robustness of findings to missing data assumptions and alternative model specifications.

#### Secondary analyses

Secondary outcomes will be analysed using the same cluster-level regression approach. Additional analyses will examine:

- Heterogeneity of treatment effects across subgroups (age, socioeconomic status, baseline HIV risk)
- Network saturation and reach characteristics using social network analysis methods
- Correlation between network position/characteristics and individual outcomes
- Cost per outcome achieved for economic evaluation

A detailed statistical analysis plan will be finalized before data lock and made publicly available.

##### Process Evaluation

A comprehensive mixed-methods process evaluation will be conducted to understand implementation, mechanisms of action, and contextual factors affecting outcomes. The evaluation follows the UK Medical Research Council guidance for complex interventions [30] and the Exploration Preparation Implementation Sustainment (EPIS) framework [31].

#### Iterative Prototyping

Prior to the main trial, the intervention will be iteratively prototyped and co-adapted through a multi-method qualitative study. The draft intervention will be piloted in two phases with a small number of primary distributors, who will be trained to deliver oral HIV self-test kits to peers and encouraged to link them to confirmatory testing and PrEP services, with recruited peers offered the opportunity to enrol as secondary distributors for a further wave of distribution. Qualitative data will be collected through forum theatre workshops, a Community Advisory Board meeting, in-depth interviews, focus group discussions, and field observational reports, with participants including men with and without intervention experience, healthcare workers, community hub operators, study implementers, and advisory board members. Findings will inform iterative adaptations across four domains, namely participant recruitment, information dissemination, incentive structures, and structural and socio-cultural factors, which will be incorporated into the final trial intervention design.

#### Pre-intervention context analysis

Prior to intervention rollout, two workshops with each of the three key stakeholder groups (men from the target population, healthcare providers, and implementers) will be conducted to refine intervention procedures and identify local resources. Four focus group discussions with men will explore social network structures, attitudes, and practices to optimise intervention reach. Participant observation will document planning processes. These activities will map barriers and facilitators for implementation and sustainability.

#### Real-time implementation monitoring

During implementation, real-time feedback will support intervention delivery optimisation. Four focus group discussions and eight individual interviews with men, six interviews with healthcare providers, and four interviews with implementers will identify barriers, facilitators, determinants of effectiveness, and unintended consequences. Anonymous feedback mechanisms, comprising a designated helpline and suggestion mailboxes, will capture staff insights. Participant observation of intervention activities and community interactions will monitor progress. Quantitative monitoring data on enrolment and cascade progression will be triangulated with qualitative findings.

#### Post-trial evaluation

The post-trial process evaluation will examine the contextual conditions and mechanisms through which the intervention produced its effects, drawing on 24 interviews and eight focus groups with diverse stakeholders. Analysis will explore contextual factors and social network mechanisms shaping experiences and engagement across the HIV prevention cascade, examining network structures, implementation processes, spatial and contextual factors, timing dynamics, and the relational norms influencing outcomes.

#### Photovoice methodology

Twenty men from intervention clusters will participate in photovoice exercises to capture lived experiences and perspectives on intervention barriers and facilitators. Following training on photography and research ethics, participants will spend two weeks photographing their responses to three guided questions: how the intervention affected them, what they considered to be its most significant accomplishments, and which aspects were ineffective or unsuccessful. Participants will then select six photographs that they feel best represent significant changes and will provide written reflections on their selections. Follow-up interviews will subsequently be conducted to explore how participants' social networks influenced their experiences of the intervention.

Ethical safeguards include comprehensive training on consent and privacy, community leadership involvement and oversight, regular photograph review, and immediate complaint reporting mechanisms. All participants will receive reimbursement for their time and contribution. Workshop participants will receive 10USD, participants in interviews or focus groups will receive two bars of laundry soap, and photovoice participants will receive a t-shirt and 10USD.

Qualitative data will be analysed using thematic analysis, with coding frameworks developed iteratively. Analysis will identify patterns across data sources and generate explanatory models linking context, mechanisms, and outcomes.

#### Mathematical modelling and cost-effectiveness analysis

PopART-IBM, an established individual-based model of HIV transmission previously used for the HPTN 071 trial [32], will be adapted to project long-term population-level impact. The model will be modified to explicitly include HIVST as a testing channel and PrEP initiation parameterised using age-specific uptake rates from trial data. Model parameterisation will incorporate HIV prevention cascade barriers identified in this population.

Using a Bayesian inference framework, multiple simulations consistent with observed trajectories will be generated to account for parametric and stochastic uncertainty. Impact will be calculated relative to simulated counterfactuals without intervention and for HIVST alone. Outcomes include HIV infections averted, deaths averted, and disability-adjusted life years (DALYs) averted under sustained uptake assumptions. Uncertainty bounds will be generated from multiple simulations.

Cost-effectiveness analysis will use actual intervention costs and observed outcomes to project benefit streams for participants, partners, and populations. Incremental cost-effectiveness ratios will be calculated and compared to established willingness-to-pay thresholds for Zimbabwe.

### Who will be included in each analysis {27b}

The primary analysis will be a complete-case analysis assuming data are missing completely at random; a sensitivity analysis using multiple imputation (assuming data are missing at random) will be performed; further details about this analysis will be included in the Statistical Analysis Plan. Complete case analyses will include all participants with full baseline and exit data.

### How missing data will be handled in the analysis {27c}

#### Missing data

Missing data patterns will be described and examined for associations with baseline characteristics and treatment allocation. Primary analysis will use complete case analysis. Sensitivity analyses will use multiple imputation methods under missing at random assumptions to assess robustness of findings.

### Methods for additional analyses (e.g. subgroup analyses) {27d}

#### Interim analyses {28b}

Interim analyses to indicate trial progress and outcomes using both blinded and unblinded data will be prepared for the trial Data Safety Monitoring Board and presented at the annual meetings and other ad-hoc meetings. The DSMB will advise the Trial PI’s if there are reasons due to safety concerns or futility which indicate the trial should be terminated early.

### Protocol and statistical analysis plan {5}

The trial protocol will be made publicly accessible through publication. The statistical analysis plan (SAP) will be available as an attachment to the trial registration and will also be submitted as an update to the published protocol paper prior to database lock.

## Oversight and monitoring

### Composition of the coordinating centre and trial steering committee {3d}

The Study Management Group (SMG) serves as both the coordinating centre and trial steering committee for the IMPERATIVE trial. It comprises the principal investigators, co-investigators, and the Zimbabwe-based trial managers. The SMG holds responsibility for decisions regarding trial continuation and protocol amendments, with proposals developed by the Principal Investigators. The trial managers oversee day-to-day operations, coordinate participant follow-up, and report to the SMG on a monthly basis. The SMG Chair receives independent oversight advice from the Data and Safety Monitoring Board (DSMB).

### Composition of the data monitoring committee, its role and reporting structure {28a}

Safety monitoring is provided by an independent Data Safety and Monitoring Board (DSMB), which operates independently of both the sponsor and funder. All members serve in an individual capacity and have declared no conflicts of interest. The DSMB membership includes senior academics and public health leaders with relevant expertise: a Professor of Medical Social Sciences and Behavioral Sciences and Director of the Ryan Family Center for Global Primary Care; an Assistant Professor in the Cancer Prevention Program at the Fred Hutchinson Cancer Center and affiliate Assistant Professor in the Department of Global Health at the University of Washington; the Director of the AIDS and TB Programme at the Ministry of Health and Childcare, Zimbabwe; and the Zimbabwe National AIDS Council’s Programme Coordinator for Mutasa District, Manicaland.

The DSMB's primary role is to safeguard participant interests and uphold the integrity, scientific validity, and credibility of the trial. To this end, the DSMB will review participant safety, study conduct, data quality and completeness, and overall trial performance, including assessment of protocol deviations as they relate to participant safety and scientific integrity. Safety monitoring will encompass evaluation of risks inherent to study participation and the potential impact of any protocol changes on participant risk. Recommendations arising from DSMB review will be communicated to the SMG Chair.

Scheduled DSMB meetings will be supplemented by ad hoc meetings where concerns regarding safety or trial progress arise, such as an unexpected frequency of serious adverse events (SAEs). Local trial teams may also refer any SAE of concern directly to the DSMB Chair. In addition, SAEs will be reported to the relevant institutional oversight bodies: the Medical Research Council of Zimbabwe Research Ethics Committee and the Stellenbosch University Research Ethics Committee, South Africa.

### Frequency and plans for auditing trial conduct {29}

Study progress and safety will be reviewed monthly throughout the intervention period, with more frequent review undertaken as required. Monthly progress reports covering participant recruitment, retention, attrition, and adverse events will be submitted to the Study Management Group (SMG). An Annual Report will additionally be compiled and submitted to the SMG, all Institutional Review Boards, and other applicable oversight bodies, addressing whether adverse event rates are consistent with pre-study assumptions, reasons for participant dropout, confirmation that entry criteria were met, and whether continued data collection remains necessary to accomplish the stated study aims. The trial may be terminated prematurely if the intervention is associated with adverse effects that call the safety of participants into question, if recruitment or retention difficulties materially compromise the ability to evaluate the study endpoints, or if new information or other circumstances arise that warrant early cessation.

### Protocol amendments {31}

Any modifications to the protocol that may impact study conduct, participant safety, or scientific integrity will be documented in protocol amendments. Amendments will be submitted for ethics committee approval and trial registry updates as appropriate. Participants will be informed of relevant changes. All protocol amendments will be registered on ClinicalTrials.gov (NCT06370923) as required under the terms of the NIH funding award

### Dissemination policy {8}

Study results will be disseminated through multiple channels. Findings will be submitted for publication in peer-reviewed open-access journals and presented at international scientific conferences. Policy briefs will be prepared for the Zimbabwe Ministry of Health and Child Care and the National AIDS Council. Results will also be shared directly with study participants and community leaders through community meetings, and the trial registry entry on ClinicalTrials.gov will be updated accordingly. De-identified datasets will be deposited in publicly accessible repositories to facilitate data sharing.

Authorship will follow the guidelines of the International Committee of Medical Journal Editors (ICMJE). All investigators who have contributed substantially to study design, data collection, analysis, or manuscript preparation will be eligible for authorship. Any use of professional medical writers will be acknowledged. Study findings will be shared with participants and communities irrespective of whether results are positive, negative, or inconclusive.

## Discussion

This cluster randomised controlled trial will provide the first large-scale evidence on whether peer network-based HIVST distribution combined with mobile health support and enhanced risk perception can increase PrEP uptake among men in a high HIV-prevalence setting. The study addresses a critical gap in HIV prevention by integrating proven individual strategies into a comprehensive, scalable intervention targeting multiple barriers men face in accessing prevention services.

This study has several key strengths. The cluster randomised design provides rigorous causal inference with adequate statistical power, and implementation within a well-characterised population with 30 years of HIV surveillance data offers an unusually robust contextual foundation. A comprehensive process evaluation will be conducted to examine the mechanisms and contextual factors underlying the intervention's effects, complemented by objective biomarker verification of PrEP adherence. Mathematical modelling will be used to project the long-term population impact and cost-effectiveness of the intervention. Finally, the study has been developed in close partnership with national health authorities, ensuring alignment with existing HIV programmes and facilitating potential future scale-up.

The study also has limitations. Cluster randomisation prevents individual-level blinding, potentially introducing bias if awareness of allocation affects behaviour. However, objective outcome measures and assessor blinding will minimise this risk. Retention challenges may occur due to the mobility of participants in this setting. To mitigate this we will implement intensive follow-up protocols and retention incentives. The intervention requires functional mobile phone networks and clinic infrastructure, which may limit generalisability to settings without these resources. Finally, we cannot assess whether individuals remain adherent to PrEP beyond six months. This will be explored through modelling projections.

If effective, this intervention offers several advantages for potential scale-up. The peer network distribution model leverages existing social structures without requiring extensive new infrastructure. Mobile phone support is increasingly feasible across sub-Saharan Africa given high mobile penetration rates. Integration with existing HIVST and PrEP programmes minimizes additional burden on health systems. The implementation science approach ensures the intervention is designed for real-world delivery, not just research conditions.

This trial will provide important evidence to inform HIV prevention policies in Zimbabwe and across sub-Saharan Africa. Positive results could support national adoption of peer network HIVST distribution with mobile support as a strategy to engage men in prevention. Even null or negative results will provide valuable insights into barriers that remain unaddressed by current approaches, guiding future intervention development. The comprehensive process evaluation will illuminate why the intervention did or did not work, under what conditions, and for whom, essential knowledge for adapting interventions to diverse contexts.

Achieving UNAIDS targets and eliminating HIV as a public health threat requires substantial improvements in male engagement with prevention services. This study tests an innovative, theoretically grounded, and potentially scalable approach to addressing this challenge. Results will advance our understanding of how to harness peer networks and mobile health technologies to reach men who remain underserved by current HIV prevention programmes.

## Figures and Tables

**Table 1:** Characteristics of individuals included in the study based on PRO EDI (Participant Reporting of Outcomes with Equity, Diversity and Inclusion) requirements

Characteristic	The people we would expect to see included
Age	18 years and above
Sex	Male
Gender	Men
Race, ethnicity and ancestry	Black African (Ndebele and Shona)
Socioeconomic status	Small towns, agricultural estates, roadside settlements, subsistence farming areas, and urban areas
Geographic location	Manicaland Province, Eastern Zimbabwe
Other characteristics relevant to the trial	HIV prevalence remains high, peaking at 27% for women aged 45–49 years and 28% for men aged 50–54 years.

**Table 2:** Participant timeline: Schedule of enrolment, interventions, and assessments.

Intervention arm		Control arm		Outcome measures (inter-arm comparison)
Time	Activity	Time	Activity	
0 days (T0)	Receipt of kit packs by distributors			
0–7 days	HIVST kit distribution to peers			
7 days – 1 month	Initial contact with recipient by helpline and Study enrolment			
<= 3 months after HIVST receipt from distributor (T1)	Visit of Recipient to primary care clinic	(T1)	Study enrolment on attendance at clinic	**Primary** Proportion initiating PrEP **Secondary** i. Proportion initiating ART ii. Proportion initiating ART or PrEP iii. Proportion taking a clinic HIV test iv. Proportion initiating ART v. Proportion initiating ART or PrEP vi. Proportion taking a clinic HIV test diagnosed HIV positive vii. Proportion taking a clinic HIV test following HIVST in past 3 months
T1 + 1 month	Monthly follow-up of participants on PrEP/ ART by helpline	T1 + 1 month	Monthly follow-up of participants on PrEP/ ART using clinic records	**Secondary** viii. Proportion initiating ART returning for first follow-up visit ix. Proportion initiating PrEP returning for first follow-up visit
T1 + 2 months		T1 + 2 months		
T1 + 3 months		T1 + 3 months		
T1 + 4 months		T1 + 4 months		
T1 + 5 months		T1 + 5 months		
T1 + 6 months	Monthly follow-up of participants on PrEP/ ART to assess adherence by study helpline & dry blood spot collection	T1 + 6 months	Monthly follow-up of participants on PrEP/ ART using clinic records	

## Data Availability

De-identified data will be made available to the research community upon reasonable request after final outcomes and subsequent analyses have been published, subject to data sharing agreements protecting participant confidentiality. Due to the sensitive nature of data collected, including information on HIV status, treatment and sexual risk behaviour, the Manicaland Centre for Public Health does not make full analysis datasets publicly available.

## Abbreviations

ART: Antiretroviral therapy
DALYs: Disability-adjusted life years
HIV: Human immunodeficiency virus
HIVST: HIV self-testing
HPC: HIV prevention cascade
ICC: Intra-cluster correlation coefficient
PrEP: Pre-exposure prophylaxis
RCT: Randomised controlled trial
SMS: Short message service
SPIRIT: Standard Protocol Items:Recommendations for Interventional Trials
SSA: Sub-Saharan Africa
TFV-DP: Tenofovir diphosphate
CAB: Community Advisory Board
CHW: Community Health Worker
GCP: Good Clinical Practice
SMG: Study Management Group
SAP: Statistical Analysis Plan
HPC: HIV Prevention Cascade
